# Integrated knowledge translation guidelines for trainees in health research: an environmental scan

**DOI:** 10.1186/s12961-023-01024-3

**Published:** 2023-07-14

**Authors:** Sarah Madeline Gallant, Christine Cassidy, Joyce Al-Rassi, Elaine Moody, Hwayeon Danielle Shin, Shauna Best, Audrey Steenbeek

**Affiliations:** 1 School of Nursing, Dalhousie University, Halifax, NS Canada; 2 The Strengthening Transitions in Care Lab, IWK Health, Halifax, NS Canada; 3 Nova Scotia Health Authority, Halifax, NS Canada; 4 IWK Health, Halifax, NS Canada; 5 Management, and Evaluation, Institute of Health Policy, University of Toronto, Toronto, ON Canada; 6 Centre for Complex Care Interventions, Centre for Addiction and Mental Health (CAMH), Toronto, ON Canada

**Keywords:** Integrated knowledge translation, Graduate students, Health systems research, Partnership research, Research collaboration

## Abstract

**Background:**

Collaborative health research, such as integrated knowledge translation (IKT), requires researchers to have specific knowledge and skills in working in partnership with knowledge users. Graduate students are often not provided with the opportunity to learn skills in how to establish collaborative relationships with knowledge users in the health system or communities, despite its importance in research. The objective of this environmental scan is to identify available guidelines for graduate trainees to use an IKT approach in their research.

**Methods:**

We conducted an environmental scan with three separate systematic searches to identify guidelines available to support graduate students in engaging in an IKT approach to research: (i) a customized Google search; (ii) a targeted Canadian university website search; and (iii) emails to administrators of graduate studies programmes asking for available guidelines and documents designed for graduate students. Data were extracted using a standardized data extraction tool and analysed using a directed content analysis approach. Due to the minimal results included based on the a priori eligibility criteria, we returned to the excluded records to further review the current state of the environment on trainee support for IKT research.

**Results:**

Our search strategy yielded 22 900 items, and after a two-step screening process with strict inclusion criteria three documents met the eligibility criteria. All three documents highlighted the need for an IKT plan for knowledge user involvement throughout the research process. Furthermore, documents emphasized the need for tangible steps to guide graduate students to engage in effective communication with knowledge users. Due to the lack of documents retrieved, we conducted a post hoc content analysis of relevant IKT documents excluded and identified five themes demonstrating increased education and engagement in an IKT approach at an interpersonal and organizational level.

**Conclusion:**

We identified three documents providing guidance to trainees using a collaborative approach in their health research. This scan highlighted two key findings including the importance of supporting trainees to engage knowledge users in research and preparing an IKT plan alongside a research plan. Further research is needed to co-design guidelines to support graduate students and trainees in engaging in an IKT approach.

**Supplementary Information:**

The online version contains supplementary material available at 10.1186/s12961-023-01024-3.

## Introduction

Collaborative research approaches, such as co-production, co-design, engaged scholarship and integrated knowledge translation (IKT), aim to bridge the evidence to practice gap, and subsequent policy gaps [1]. IKT, specifically, is defined by Kothari and colleagues as “a model of collaborative research, where researchers work with knowledge users (i.e. patients, families, clinicians, decision-makers) who identify a problem and have the authority to implement the research recommendations (p. 299)”[2]. IKT has the potential to improve the quality of research completed [3], enhance the value of the research for decision-makers [4], improve the capacity of key knowledge users to engage meaningfully in research [4–6] and yield more useful results that provide greater impact to those affected [7–10].

Despite efforts to promote collaborative research approaches, most health research continues to operate independently from the health care system [11]. Key knowledge users, decision makers and other individuals impacted by research evidence are often left out of the research process. This fragmented and disconnected approach to research has led to challenges in ensuring successful completion of research projects, and implementation of meaningful changes based on research findings [11]. Several barriers to collaborative health research have been reported at the individual, interpersonal and organizational levels[12]. At the individual level, IKT is resource intensive, requiring a specific set of skills, knowledge and sufficient time [7]. Further, at the interpersonal level, establishing and maintaining research partnerships with key knowledge users can pose a significant barrier in successfully using an IKT approach [4, 7, 13]. A lack of understanding and skills of the collaboration process are significant barriers to the success of research partnerships with key knowledge users [4]. Lastly, the organizations that support trainees, knowledge users and researchers often have competing priorities (e.g. timely thesis/project completion, patient care, service delivery, resource use) which make it challenging to engage in IKT research [12, 14].

To address these barriers, effective collaborative health research requires researchers to have specific knowledge and skills to work in partnership with knowledge users [15]. Although there is a growing emphasis on researchers establishing knowledge and skills in collaborative partnerships, traditionally, IKT has not been taught in graduate health research programmes [15]. A survey of PhD-prepared researchers showed that they have unmet learning needs related to collaboration in research during their training [16]. Graduate students are often not provided with the opportunity to learn skills on how to establish collaborative relationships with knowledge users in the health system[13], and most do not receive training on collaborative health research approaches [17, 18]. Further, health system leaders report a significant lack of preparation in graduate health students engaging in collaborative research partnerships [19], which impedes the ability for students to successfully engage in an IKT approach to their research. Graduate students in health-related programmes should have access to resources to support their successful development of knowledge and skills in creating collaborative partnerships with key knowledge users in research [12].

We recently conducted a scoping review to identify how trainees have used an IKT approach in their health research [12]. A trainee is defined by the Canadian Institute of Health Research (2023) as “an individual who is enhancing their research skills through actual involvement in research and who works under the formal supervision of an independent researcher (p. 1)” [20]. The review identified a need for increased graduate level education and skills in conducting IKT, and a need to promote the value of IKT in trainee-led research [12]. Specifically, one major finding was that trainees reported a lack of knowledge and skills on co-production and difficulty navigating multiple competing priorities with their knowledge users. Despite the lack of knowledge and skills, trainees across diverse disciplines (e.g., nursing, physiotherapy, medicine, education) showed an overwhelming interest in using an IKT approach to research [12]. Currently, trainees who engage in research partnerships with knowledge users are often self-motivated, supported with experiential learning opportunities, and are supervised or receive mentorship from established researchers with expertise in IKT [21]. Establishing effective skills in building trusting, collaborative partnerships with knowledge users is imperative to ensure all health research trainees are engaging in meaningful, ethical research with relevant outcomes [22].

Efforts are needed to improve academic preparation for engaging in health research partnerships [19, 21]. A guideline is a promising tool to address the identified gap in knowledge and skill development for trainees in health research. Guidelines are familiar documents used in healthcare to support education and training of healthcare professionals [23]. As such, the objective of this environmental scan is to identify available guidelines and/or resources for graduate students and trainees to use an IKT approach in their research. To our knowledge, no reviews have been completed on this topic. Results from this review will guide the development of guidelines to support trainees in engaging in an IKT approach to research, thus improving the relevancy and impact of their research.

This paper reports on phase one of a multiphase study that aims to co-design guidelines for engaging in an IKT approach in graduate studies. The research team has engaged in an IKT approach in the completion of this study. The team is comprised of graduate students (lead author, and author 5), undergraduate student (author 3), health system partner (author 6) and graduate program educators (authors 2, 4 and 7). The larger program of work aims to address the following research question: How can graduate students use an IKT approach in their thesis work? As a first step in this study, we conducted an environmental scan of relevant documents related to guiding graduate students to engage in an IKT approach to research [24].

## Methods

We conducted an environmental scan, a systematic approach to exploring the available information on a specific topic, to gain an understanding of the current state of resources available for graduate students in health to engage in an IKT approach to research [25]. The environmental scan followed Godin’s grey literature search methodology [26]. This methodology encompasses complementary search strategies including (1) customized Google searches, (2) a targeted website search and (3) expert consultations [26].

### Aim of the environmental scan

The aim of the environmental scan was to identify and review relevant documents available to guide graduate students in engaging in an IKT approach to research. The following question and sub-question were used to guide the scan.

- 1. What guidelines exist for graduate students to use an integrated knowledge translation approach in their research?
- A. What are key components of the guidelines to be included in future co-development of guidelines for trainee engagement in IKT?

### Identify relevant resources

We outlined key inclusion criteria in the categories of population, concept and context, to ensure identification of relevant studies [27]. Inclusion criteria has been developed on the basis of our previous work which identified a lack of guidelines for graduate students in health to engage in an integrated knowledge translation approach to health research [12]. We included only documents in the English language as authors were fluent in only the English language, which limited the ability to adequately assess documents completed in another language. No limits were set on publication date to document the evolution of documents over time.

#### Population

We included resources developed for the target audience of graduate students in health research. A graduate student can include masters’, doctoral or post-doctoral students in health-related research programmes. We also included resources that are noted for research trainees in health as defined by the CIHR as “an individual who is enhancing their research skills through actual involvement in research and who works under the formal supervision of an independent researcher (p. 1)” [20].

#### Concept

We included all documents, guidelines, papers and/or resources, hereby referred to as documents, that provided guidance on how to engage in collaborative partnership research, including but not limited to integrated knowledge translation, research co-production or engaged scholarship research. These approaches share similar underlying purposes of conducting research with the intent to enact change by engaging in collaborative partnerships with key knowledge users [1]. For the purpose of this review, authors have focused on IKT as the collaborative research term, given the context of the research is geographically located in Canada, where IKT is the most commonly used term, and origins are located in the discipline of health [1].

#### Context

This environmental scan will include documents developed for graduate students in health-related programmes.

### Search strategy

We completed three complementary search strategies following Godin’s methodology. We worked with a librarian scientist to identify keywords to be used in the searches. We completed the environmental scan between April 2022 and August 2022. We screened the potential resources using the above-listed inclusion criteria to ensure relevant resources were included to meet the outlined research question and sub-question. We took a two-step screening approach to each customized search strategy. First, we completed a scan of the results based on their title and supplemental text; eligible materials based on title were saved in PDF form. Second, all eligible saved PDFs were screened on the basis of outlined inclusion criteria. Two reviewers met regularly to discuss the screening process and documents reviewed. A third reviewer (author 2) addressed any arising discrepancies between reviewers. We saved all documents meeting the outlined inclusion criteria.

### Part 1: customized Google search

We conducted a customized search based on the power of relevancy ranking within the Google search engine to bring the most relevant results to the top of the list [26]. Then we pre-determined the number of pages we screened to ensure feasibility and consistency across searches. We used the Google search engine in “incognito” mode to ensure no recommended websites were in the search due to personal history. We completed six separate searches based on the keywords determined in consultation with the librarian specialist. The following key phrases were used in each search (1) “Integrated Knowledge Translation” Guide; (2) “Co-design” “In research” Guide; (3) “Knowledge User Engagement” “in research” Guide; (4) “Co-Production Research” Guide; (5) “Engaged scholarship” “in research” guide; and (6) “Collaborative Research Approach” Guide.

We first conducted a Google search using the above-outlined search phrases to identify the relevant organizations and websites publishing documents on the relevant subject area. The team reviewed the first 10 pages of each search’s hits (representing 100 results) for potentially relevant titles (supplemented with the text under the title). We recorded the website’s name/organization and Uniform Resource Locator (URL) into an Excel spreadsheet of potential documents meeting the inclusion criteria (Table 1). We saved the URLs to be further screened by two reviewers.

**Table 1:** Inclusion criteria template and associated inclusion definitions

Population (graduate students/trainees)	Documents must include: (a) Acknowledgement that the document can be used by “trainees” including graduate students or postdoctoral researcher trainees (b) Can include documents that note the resource is for “researchers” if there is a definition including trainees defined in that title
Concept (a guide for graduate students/ trainees to engage in IKT research)	Type of “documents” to include: (a) The document must provide guidance specific to trainees in engaging in an IKT approach (or any other collaborative research approach) to research (b) Documents can be in any format such as (i) a paper, (ii) a resource or (iii) a guideline, as long as the source is providing a guide (c) Guide is defined as providing steps, knowledge or instruction on how to engage in an IKT approach to research
Context (health research programme)	Document must be designed for supporting IKT (or other collaborative research approaches) in a health research programme

Next, we hand-searched each of the relevant websites’ homepage for potentially relevant documents (e.g. web pages, reports). Within this step, we documented each website and the date each search was completed. Two reviewers screened all applicable documents and resources using a standardized inclusion template (Table 1). The documents meeting the inclusion requirements were kept for data extraction.

### Part 2: Targeted Canadian university website search

One reviewer (author 1) completed a Google search to determine all possible Canadian universities that offered graduate programmes in health (*N* = 45). We chose to limit the targeted university website search to Canadian universities because the aim of the proposed environmental scan was to review what guidelines existed for trainees in health research to guide part 2 of the study. In part 2 of the study, we will be engaging in the co-design of guidelines for trainees’ engagement in IKT in a Canadian university; therefore, it was important to find documents which were context specific to Canadian universities. Furthermore, understanding IKT in the Canadian post-secondary system was imperative to ensure we were reviewing resources within the Canadian geographic, allowing us to use findings to inform future work. In addition, it was beyond the scope of the project to do a targeted search of all universities in the world. Further, documents from non-Canadian universities would be captured in the Google search. We used the search function on the qualifying university websites to search for each of the following terms: (1) “Integrated Knowledge Translation Guide”; (2) “Co-design guide”; (3) “Knowledge user engagement guide”; (4) “Co-Production Research Guide”; (5) “Engaged scholarship guide”; (6) “Collaborative Research Approach guide”. We reviewed the first 100 results from each individual search. The university websites use Google as a search engine for their website; therefore, the power of relevancy was assumed in the first 100 results.

We engaged in the two-step screening process as described above to identify relevant documents to meet our outlined research question and sub-question. Throughout the screening process we documented key aspects of the search including (1) the keywords used, (2) date searched, (3) total results retrieved, (4) an email contact (for search three detailed below) and (5) a copy of the eligible documents in PDF form. The documents saved were included for data extraction.

### Part 3: Consultations with administrators via email

During the previous searches, we noted the email address of any expert (e.g. university administrators) to include in the final search. We saved a total of (*N* = 45) emails and sent an initial email on 23 June 2022 to prospective graduate student administrators. The email outlined the purpose of the proposed project and requested graduate student administrators to send any known documents available at their perspective universities that could be used as a guide for trainees to engage in an IKT research approach. Emails were sent directly to the Associate Dean of graduate studies where available, and if not available, an email was sent to the graduate studies email.

We sent the email using a secure university account (author 1). We documented the date the email was sent, responses, documents received and any follow-up. We were open to receiving responses up to 2 months following the sent date. Follow-up emails were sent where deemed necessary (e.g. another contact was provided).

We engaged in the two-step screening process as described above to identify relevant documents to meet our outlined research question based on the material that was sent through email. The documents meeting the inclusion requirements were kept for data extraction.

### Data extraction

We created a data extraction tool to collect general information on the documents that met all three inclusion criteria, in three specific categories. First, we captured general information on the document’s characteristic including author, type of author, year of publication, purpose of document, setting, location and format of the document. Next, we identified characteristics of the documents that were specific to graduate students, including the following topics: health discipline, stage of training, and type of programme the document was geared towards. Finally, we reviewed all IKT content that was included in the document including a description of (1) the type of knowledge users involved; (2) the level of engagement based on the International Association of Public Participation (IAP2) tool [28]; (3) the steps taken to engage in an IKT approach; (4) suggested timeline to engage knowledge users in the research process; (5) reported outcomes of using an IKT guideline; and (6) any implications or recommendations. We piloted the data extraction tool with two independent reviewers (authors 1 and 3). No modifications were made following the pilot testing. We extracted data using an Excel spreadsheet, and a third reviewer (author 2) addressed any discrepancies between reviewers.

### Data analysis

We used a directed content analysis approach [29] to analyse the extracted data from the documents meeting the inclusion criteria. Specifically, we coded the data using the International Association of Public Participation (IAP2)to determine how the documents suggested involving knowledge users in an IKT approach to research [28]. The IAP2 provides a framework to guide public participation in research endeavours. It encompasses a spectrum from “least” involved to “most” involved the public can be on research projects. The spectrum includes the following categories from least to most involved; (1) inform, where the public is informed of the project; (2) consult, where the researcher elicits feedback from the public; (3) involve, where the public is involved in the research process; (4) collaborate, where the public is involved in decision-making processes for the project; and (5) empowerment, where the public makes the final decisions for research processes [28]. Theoretically, only the collaborate and empowerment levels should be considered IKT; however, we used the full spectrum to understand how IKT is being described and employed in the documents.

Furthermore, we examined the stages of research that trainees were encouraged to engage knowledge users in, using Greenlee et al. engagement in the research process categories [30]. Finally, we narratively synthesized a description of outcomes explicitly shared in the document along with the content related to IKT.

### Post hoc conventional content analysis

Due to the minimal retrieval of documents meeting the a priori eligibility criteria, we returned to the excluded records originally screened out during the second stage of the screening process for part one through three (*N* = 176). We felt there may be important insights to gather from the excluded records that were relevant but did not meet all three inclusion criteria. We included any document that was screened in during the first screening step.

#### Data extraction

We took field notes throughout the second stage of screening including listing the (1) content type (i.e. educational material, IKT event), and (2) reason for exclusion (i.e. resource was not inclusive for trainees) of the documents excluded in the second stage.

#### Data analysis

We used a conventional content analysis approach to further categorize the extracted data on the records excluded from the second stage of screening. We engaged in inductive category development with this dataset through back-and-forth discussion amongst team members [29]. First, we read all the extracted data repeatedly to fully immerse ourselves within the content. Next, we derived potential codes from the data extracted. Finally, we organized the codes into categories to create meaningful themes representative of the data found [29].

## Results

**Fig. 1:**
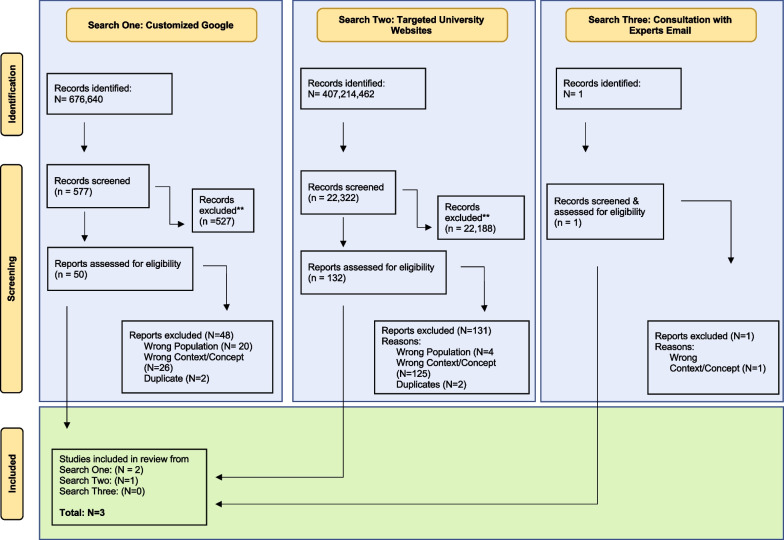
Search diagram adapted from: Page MJ, McKenzie JE, Bossuyt PM, Boutron I, Hoffmann TC, Mulrow CD, et al. The PRISMA 2020 statement: an updated guideline for reporting systematic reviews. *BMJ* 2021;372:n71. https://doi.org/10.1136/bmj.n71

Our environmental scan yielded a total of 22 900 items. After initial screening of titles, 183 resources remained for assessment based on detailed inclusion criteria. After second stage screening and removing duplicates (*N* = 4), three resources were included in the final review (Fig. 1). We report each search below separately. A summary of the results is included in Table 2**.**

**Table 2:** Results of three documents retrieved from the individual searches including (1) customized Google search, (2) targeted university search and (3) consultation with administrators

Included document	General characteristics	IAP2 level of engagement [22]	Engagement in research process [23]	Summary of IKT guideline content
Involving consumers in health and medical research [32]	Publication year: 2021 Located: customized Google search Format: PDF format	Collaborate	Engagement from project conception; throughout entire research process	• Guidelines were created in partnership with a health organization and knowledge translation service • Knowledge users were defined as anyone who may be affected by research • Encouraged a detailed plan for IKT • Encouraged an agreement document be signed by all parties involved • Identified the concept of a “research mentor” • Reported outcomes: improved relevancy and effectiveness of proposed research
A manual for community based participatory research [31]	Publication year: 2013 Located: customized Google search Format: PDF format	Collaborate	Engagement from project conception; throughout entire research process	• Guidelines were created in partnership with a health organization and a university • Knowledge users were defined as anyone who may be affected by research • Defined IKT engagement planning as a cyclical process • Reported outcomes: improved relevancy and effectiveness of proposed research
University of Alberta Student Participation Process Handbook [33]	Publication year: 2015 Located: targeted university website Format: PDF format	Engagement	Variable depending on the purpose of the engagement of the knowledge user	• Knowledge users are defined on the basis of the impact the decision/project has on an individual • An emphasis on including an engagement plan • No cited outcomes to using the resource

### Part 1: Customized Google search

In total, 577 results were reviewed from the customized Google search and 50 potential resources were identified for full-text review. Of these, only two documents [31, 32] met the inclusion criteria. Neither of these documents was written directly for graduate students/ trainees; however, they included a category of researchers, including students, as part of the description. Both documents aimed to provide direction for researchers to engage in an IKT or similar type of collaborative research approach. Both documents were created in partnership: one document was a partnership between The Center for Excellence in Assisted Living (a health organization) and University of South Carolina (a university) [31], while the other was a partnership between Australian Health Research Alliance (a health organization) and Western Australian Health Translation Network (a knowledge translation institution) [32]. Documents were developed in the United States (*N* = 1) and Australia (*N* = 1). One document was developed in 2013 [31], while the other was more recent in 2021[32]. Both documents were accessible online through a Google search in PDF format.

In terms of IKT content for both documents, knowledge users were described as any consumer or person who would be affected by research [31, 32]. This statement was purposefully broad to include all possible consumers of research including patients, health organizations, funding agencies, community members, etc. Both documents emphasized the importance of knowledge users being involved in all aspects of the research, with a clear recommendation for knowledge users to be decision makers in the research process. Due to the emphasis and focus on decision-making, we classified both documents on the IAP2 scale as collaborate [28].

Both documents describe steps to ensure a seamless IKT approach, including a cyclical planning process [31, 32]. Both documents emphasized the importance of developing a plan for IKT, including a detailed step-by-step engagement and communication plan to ensure successful engagement throughout the research design. Furthermore, in applying a research process lens, as described by Greenlee et al. to the engagement of knowledge users, both documents identified the importance of early engagement from the project conception and planning stages [30]. Finally, both documents outlined the potential outcomes of using their resource to improve relevance and effectiveness of proposed research by using an IKT approach to research [31, 32].

### Part 2: Targeted university search

In total 22 322 results were reviewed from the targeted university search; from these, 132 were identified as potential documents guiding graduate trainees in using an IKT approach to research. Two duplicates were removed. Of these 130 potential documents, only one met the detailed inclusion criteria [33]. The document was identified as a resource for engaging students in projects and research across a continuum. Although not directed to graduate students specifically, the document included all students at the university. The document was created in 2015 at the University of Alberta, with the intention that students and faculty use the document in university level projects and research. Ultimately, the purpose of these guidelines was to ensure diversity in participation in projects especially in terms of meaningful engagement and decision-making. This document was available in PDF format and accessible through the university’s website.

In terms of IKT content, the document described knowledge users as any individual that may be affected by the project and/or decisions made during the project/research. In applying the IAP2 [28] framework to data extraction, this document described the need to involve knowledge users on a continuum, depending on the needs of the project [33]. This recommendation is unlike the previous two resources analysed in the Google search [31, 32], as they both emphasized the importance of collaboration with all knowledge users despite the nature of the project. This document [33] emphasizes the importance of meeting the “involve level” in the IAP2 framework; however, it does not necessarily emphasize that each project must meet the collaborate or empowerment levels of engagement [28].

Furthermore, the steps to engage in an IKT approach were outlined with an emphasis on the planning stage to establish clarity of the knowledge users needed to be involved, and degree of involvement required. Additionally, there was an emphasis on planning for communication between researchers and knowledge users, ensuring there was a plan to establish and maintain effective communication throughout the project [33]. There were no specified outcomes reported in using this document in IKT research.

### Part 3: Consultation with administrators

Nine university administrators responded to our email. Only one administrator provided a document in response to our request; however, it was a guideline for researchers and not for graduate students and so was not relevant to our review. Other responses included (1) automatic responses with no follow-up (*N* = 4); (2) responses indicating that the recipient of the email was unable to provide guidance (*N* = 2); and (3) responses indicating an alternate contact to follow up with (*N* = 3). Follow-up emails were sent to the alternate contacts identified, and no responses were received with the follow-up emails. No documents from this search strategy were included in data extraction.

### Post hoc analysis: Response to minimal results

After the initial search yielded only three documents meeting a priori inclusion criteria, authors returned to the records excluded (*N* = 176) in the second stage of the screening process to conduct a post hoc analysis. Numerous documents (*N* = 71) were removed as the title originally screened was misleading and the record was not applicable to the study. A total of 105 records were included in the post hoc analysis. Five themes were identified through a conventional content analysis [29] highlighted in Table 3.

**Table 3:** Five themes identified through conventional content analysis in post hoc analysis

Theme	Frequency of documents demonstrating theme	Example from included documents
(1) Strategic plans and annual research reports with the goal of collaborative research (using IKT approaches)	Customized Google search (*N* = 1)	Concordia University • Outlined goal of engagement in collaborative research • Outlined goal of collaborative research approach since 2015 • Specifically in 2019/2020 Annual Report contained a priority initiative to engage in research partnerships abroad (engage in collaborative research projects)
	Targeted university search (*N* = 15)	
(2) Grant funding applications and resources supporting and/or requiring IKT approach in application	Customized Google search (*N* = 1)	University of Alberta; IKT and Grant Application Workshop [34] • Workshop intended for graduate students • Support in applying for a CIHR Grant • Detailed discussion on KT engagement plans and requirement for applications • Emphasis on KT engagement plans
	Targeted university search (*N* = 12)	
(3) Courses, events and education sessions for graduate student engagement in an IKT research approach	Customized Google search (*N* = 6)	University of Calgary [35] • In 2019, the University of Calgary held a symposium of mobilizing knowledge on newcomers • Event encouraged collaboration and networking amongst knowledge users and researchers • Encouraged discussion on priority topics to be addressed through research
	Targeted university search (*N* = 13)	
(4) Information or education materials emphasizing importance of using an IKT approach in research	Customized Google search (*N* = 18)	Canadian Institute of Health Research [36] • PDF document found online • Overview of integrated knowledge translation including definitions, examples and worksheets • Inclusion of a proposal worksheet for incorporating an IKT approach to health research • Emphasis on the importance of engaging in IKT to improve patient outcomes
	Targeted university search (*N* = 20)	
(5) IKT Toolkits specific to researchers, but not inclusive of graduate students/trainees	Customized Google search (*N* = 20)	Social Sciences and Humanities Research Council of Canada [37] • One-page infographic • Multistep process to successfully engage in an integrated knowledge translation approach • Purpose of the infographic is to guide partnerships
	Targeted university search (*N* = 5)	

The first theme relates to “strategic plans and annual research reports with the goal of collaborative research”. Many universities emphasized the goal of partnerships and collaboration in research (*N* = 16). Some universities went as far as outlining goals for IKT research approaches for their faculty and students, but did not include any guidelines or recommendations for graduate students.

The second theme identified was “Grant funding applications and resources supporting and/or requiring IKT approach in application”. In the screening process, we found that many grant and funding applications required or emphasized the importance of using an IKT approach in research (*N* = 13).

The third theme identified was “courses, events, and education sessions for graduate student engagement in an IKT research approach”. We found that many courses, events and education sessions for students emphasized the importance of using an IKT approach in research (*N* = 19); however, none of these results were noted to be guidelines for trainees on how to engage in IKT research.

The fourth theme identified was “Information or education materials emphasizing importance of using an IKT approach in research”. This theme was the most prominent (*N* = 38). While many of the documents had valuable IKT content and discussion, most were not tailored to graduate students, and as such, were not deemed eligible for inclusion in the environmental scan. Many of the results included resources and information sessions on IKT and the importance of including this approach to improve research outcomes; however, none of these results were specific guidelines for trainees.

Finally, the fifth theme identified was “IKT Toolkits specific to researchers, but not inclusive of graduate students/trainees”. Many resources outlined specific tool kits to be used by researchers to ensure seamless engagement in an IKT approach to their research (*N* = 25). These toolkits were not, however, tailored to graduate students.

## Discussion

We conducted an environmental scan to identify publicly available documents for graduate students and trainees in health to use an IKT approach to research. There was a lack of documents identified (*N* = 3) for graduate students and trainees to use an IKT approach to their research. The three documents meeting a priori eligibility criteria were not specific to IKT, but were designed to guide broad engagement in collaborative research methods. Although the documents were not specific to IKT, we did identify several important insights to support future work and guideline development for trainees using an IKT approach to research.

We identified two important findings from the three included documents identified in our environmental scan. These findings will be instrumental in guiding future resource development, implementation and evaluation for graduate students in health. First, all three documents emphasized the importance of engaging knowledge users in the research process [31–33]. More specifically, it was noted that engagement is critical to consider at the beginning of the project/research conception, urging graduate students/trainees to reflect on the involvement of their prospective knowledge users from project outset. Two out of the three documents suggested using a collaborative approach in all research situations [28], with an emphasis on shared decision-making amongst knowledge users and researchers as being key to successful collaborative research [31, 32]. These findings are echoed in the literature that highlights improved relevancy in results and translation of findings occurs with collaboration, empowerment and early engagement with key knowledge users [36, 38]. Early engagement of key knowledge users supports researchers to design research methods accessible and appropriate for their target population and, ultimately, improves richness and relevancy of results to improve health outcomes [38]. These findings further highlight the need to co-develop guidelines to support graduate students/trainees in health to engage in early reflection of knowledge user involvement in their research.

Second, in all three resources, there was an emphasis on including an IKT plan separate from the research proposal to ensure an IKT approach to research was implemented and sustained over the duration of the project [31–33]. When IKT engagement plans are thoughtfully developed, reviewed and evaluated throughout the research process, there is greater likelihood of improvements in research relevancy and uptake [39]. The use of an IKT plan alongside the research plan ensures engagement is sustained throughout the project [39]. We found similar findings in our environmental scan. The resources described steps related to the process of engaging and sustaining an integrated knowledge translation approach, partnering with knowledge users, and empowering them throughout the entire research process. Our previous scoping review identified that trainees reported feeling like outsiders to organizations, and cited this as a barrier in engaging in an IKT approach [12]. An IKT plan is a potential way to mitigate the feeling of being an outsider, as relationships are created at the inception of the project to build stronger collaborative research partnerships [40].

The included documents detailed communication plans on how to engage with knowledge users throughout the duration of the research project. The communication plan was made alongside the research protocol, ensuring that knowledge users would be properly engaged and empowered at every step of the research process. This included a range of activities, such as detailing monthly meetings to ensure feedback was received in a timely manner and developing an involvement agreement document to ensure all parties were aware of their respective responsibilities [32]. These activities were particularly important to ensure seamless engagement throughout the entire research process. Furthermore, one document suggested the designation of a research mentor, responsible for ensuring the inclusion and support for the knowledge user throughout the process [32]. Having a research mentor could potentially enhance the relationship between the research team and knowledge users, ensuring that the most effective outcomes can be achieved [32].

### Post hoc analysis discussion

Our previous scoping review on trainee experiences with IKT [12] revealed important barriers in using an IKT approach for trainees at the individual, interpersonal, and organizational level. Our scoping review proposed the need for a culture shift in improving infrastructure supports for IKT in trainee led research [12]. Our post hoc conventional content analysis highlighted how this culture shift is happening through the identification of two important observations at the interpersonal and organizational level.

First, we found that collaborative research is a strategic goal for many Canadian universities. This finding demonstrates how universities are starting to value an IKT approach to research by engaging with key knowledge users in the community as a means of supporting research partnerships. Several documents from the targeted university search included strategic planning and annual reports that outlined goals for IKT research in their university programmes (*N* = 16). For instance, Concordia University outlined the goal of engaging in collaborative research approaches since 2015 [41]. Similarly, the University of Calgary stated a goal of integrated and collaborative research in their strategic research plan since 2012 [42].

There has been a shift in educational opportunities and events offered at universities in recent years to improve education and knowledge in IKT. In 2019, the University of Calgary held a symposium of mobilizing knowledge on newcomers [35]. This event was designed with four main goals in mind, one being that knowledge users and researchers (including graduate students) would have a space to collaborate and discuss priority concerns from a knowledge user point of view, to be addressed in research [35]. Providing the space for discussion and partnership between knowledge users and researchers, followed by education sessions from experts on collaborative research, is an example of how change is happening to educate graduate students in IKT.

Second, our previous scoping review revealed that a lack of funding was a barrier in using an IKT approach in trainee-led research [12]. Interestingly, we found throughout our post hoc analysis several documents emphasizing the need for an IKT approach in research funding applications (*N* = 13). This finding has also been noted in recent literature [1, 7]. Globally, some funding agencies have started to require the use of an IKT approach for grant applications and recognize the impact that an IKT approach has on research outcomes and uptake of research knowledge in practice [1, 7]. Many documents, in our post hoc analysis, described funding application requirements as having a plan for engaging in IKT in the proposed research plan. For instance, during the targeted university search, Strategy for Patient Orientated Research (SPOR) support units across Canada were highlighted on university websites due to their funding opportunities for graduate students. Maritime SPOR Support Unit (MSSU), for instance, offers the “MSSU Trainee Support Program”. This application requires students to share a knowledge user engagement plan as a critical component of their research proposal. The knowledge user and patient engagement plans constitute one third of the points allotted for the award [43].

### Implications for research and practice

Despite these strategic goals, education events and IKT-related funding calls, no resources were offered by the university to guide graduate students/trainees in health research to meet this goal. Although numerous guidelines have been developed for researchers (*N* = 25), as identified in our post hoc analysis, these guidelines continue to be geared towards established researchers and not trainees. This lack of consideration for trainees leaves a gap in addressing unique barriers and challenges that trainees experience [12]. We recommend addressing this gap in the IKT literature by co-designing guidelines for engaging in an IKT approach to research. In using a co-design approach, end users (i.e. researchers, knowledge users and graduate students/trainees) are engaged creatively throughout the entire design process to ultimately improve the uptake of change in practice [44]. Furthermore, sustainability and maintenance of health care innovation and change can be improved through engagement in a co-design process [44]. Knowledge user engagement in a co-design process can address any equity concerns, along with any specific barriers to the individual [45]. Through engagement in a co-design event, guidelines for engaging in an IKT approach to research could be developed encompassing steps reflective of equity concerns and barriers found at an individual level for graduate students [12].

### Limitations

This environmental scan has several limitations. First, we developed the search strategy based on previous research completed on terminology for partnership research [46]; however, partnership research approaches vary, and it is possible that we may have overlooked guidelines using different terminology. Second, the term integrated knowledge translation is a predominantly Canadian term, and therefore there is potential we may have missed resources describing research partnership guidelines in other languages or terminologies. Third, we limited included documents to those written in the English language. Authors were fluent in only the English language, which limited the ability to adequately assess documents completed in another language. Lastly, due to resource constraints, we were only able to conduct a targeted hand search and email survey of Canadian universities. We may have missed relevant records from other international universities or colleges.

## Conclusion

This environmental scan aimed to identify the current state of guidelines for trainees to engage in an IKT approach in Canadian universities. We identified a lack of documents to support graduate students and trainees in using an IKT approach to health research. The three included documents outlined the importance of early engagement with key knowledge users, including how to properly engage and maintain relationships throughout the research process. The documents also outlined the importance of establishing an IKT plan separate from the research plan, ensuring that engagement of knowledge users was planned and evaluated throughout the research process. Although minimal documents were included, two important findings were noted in the post hoc conventional analysis of excluded records. At an organizational level, universities across Canada and funding agencies are starting to recognize the importance of using an IKT approach in trainee-led research. Universities are hosting educational events and funding agencies are offering support to graduate students engaging in an IKT approach in their research. Further efforts are now needed to build on this momentum and address barriers at an individual level supporting trainees to gain the required knowledge and skills to use an IKT approach to health research.

## Supplementary Information

**Additional file 1: Table S4.** Excluded records.

## Data Availability

The datasets used and/or analysed during the current study are available from the corresponding author on reasonable request.

## Abbreviations

IKT: Integrated knowledge translation
IAP2: International Association of Public Participation
