# Supplementary material for: Integrated knowledge translation guidelines for trainees in health research: an environmental scan
Source: Health Res Policy Syst. 2023 Jul 14;21:74. doi: 10.1186/s12961-023-01024-3 (PMC10349463; doi:10.1186/s12961-023-01024-3)
Supplement: Supplementary file 1 — Additional file 1: Table S4. Excluded records. [file 12961_2023_1024_MOESM1_ESM.pdf]

Table 4.0 Excluded Record from Second Round of Screening N=105*

*Excluded Records [N=176]
Excluded from conventional content analysis as title was misleading (i.e., record included was not inclusive of the topic inquiry) [N=71]
Duplicates noted in review [N=4]
Total of Records included in content analysis [N=105]

Record Title	Type & Reason for Exclusion	Topic (Theme)	Located at:
**Google Search**			
**1. Guide to Knowledge Translation Planning at CIHR: Integrated and End-of Grant Approaches**	Website/ Document<br><br>**Excluded:** Wrong Population context & concept	Information on IKT (4)	https://cihr-irsc.gc.ca/e/45321.html
**2. What are the IKT Guiding Principles?**	Website<br><br>**Excluded:** Wrong Population context & concept	Information on IKT (4)	https://ikt.ok.ubc.ca/

**3. Integrated Knowledge Translation in Childhood Disability…**	Website<br><br>**Excluded:** Wrong Population context & concept	Information on IKT (4)	https://www.canchild.ca/en/resources/111-integrated-knowledgetranslation-in-childhood-disability-engaging-with-partners-throughout-theresearch-process%20An

**4. Integrated Knowledge Translation Case Notes**	PDF<br><br>**Excluded:** Wrong Population context & concept	Research Example (4)	https://www.uwo.ca/fhs/kt/research_projects/2020-HS-9623-Case-Notes.pdf
**5. How we Work together**	PDF<br><br>**Excluded:** Wrong Population context & concept	Research Example (4)	https://ktpathways.ca/system/files/resources/2020-12/IKTRN-Casebook-Vol-2.pdf
**6. Guide to Knowledge Translation**	PDF<br><br>**Excluded:** Wrong Population	Guideline (5)	https://pjp-eu.coe.int/documents/42128013/47261953/PREMS+018621+GBR+2600+Guide+youth+research+WEB+16x24.pdf/7284301a-1334-0119-9300-a8114c417d45

**7. Information in Action**	Website<br><br>**Excluded:** Wrong Population context & concept	Education Session (3)	https://library.nshealth.ca/InfoinAction/GettingBuyin_ResearchCapacity
**8. Knowledge Translation**	Website<br><br>**Excluded:** Wrong Population context & concept	Education Program (3)	https://www.child-bright.ca/knowledge-translation

**9. A Researcher Guide to Knowledge Translation**	PDF<br><br>**Excluded:** Wrong Population	Researcher Guide (5)	https://cdpp.ca/sites/default/files/Guide%20to%20K nowledge%20Translation_%20Infographic.pdf
**10. Introduction to Knowledge Translation**	Website<br><br>**Excluded:** Wrong Population context & concept	Information on IKT (4)	https://mssu.ca/get-support/resources/introduction-to-knowledgetranslation/

**11. IKT Strategies for Difference Audiences**	PDF<br><br>**Excluded:** Wrong Population context & concept	KT Strategies (4)	https://www.albertahealthservices.ca/assets/info/res/mhr/if-res-mhr-ktstrategies-for-different-audiences.pdf
**12. KT Planning**	PDF<br><br>**Excluded:** Wrong Population	Guide for Researchers (5)	https://www.albertahealthservices.ca/assets/info/res/mhr/if-res-mhr-ktstrategies-for-different-audiences.pdf
**13. How to Incorporate Patient Engagement and Integrated Knowledge Translation**	Presentation<br><br>**Excluded:** Wrong Population context & concept	Grant Information (2)	https://era.library.ualberta.ca/items/05defd25-b846-43ac-a01b-6304bc834078/view/6612a827-3991-4f5d-8fca-d1f66e8ab407/2020-0204%20-%20Patient%20Engagement%20&%20iKT%20Workshop%20%20Powerpoint.pdf
**14. Knowledge Translation, Dissemination, and Impact A practical Guide for Researchers**	PDF<br><br>**Excluded:** Wrong Population	Guide for Researchers (5)	https://hseresearch.ie/wp-content/uploads/2021/04/Guide-no-1-What-isknowledge-translation-what-does-it-involve.pdf

**15. Stakeholder Engagement Guide of Guides**	PDF<br><br>**Excluded:** Wrong Population	Guide for Researchers (5)	https://www.slideshare.net/NeuroDevNet/stakeholder-engagement-guideof-guides-accessible2
**16. Guide for Embedded Co-Design**	Website<br><br>**Excluded:** Wrong Population	Guide (5)	https://wiki.fluidproject.org/display/fluid/Guide+for+Embedded+Co-design
**17. Co-Design As Collaborative Research**	PDF<br><br>**Excluded:** Wrong Population	Guide (5)	https://connected-communities.org/wp-content/uploads/2018/07/CoDesign_SP.pdf
**18. MinD CoDesign Guidelines**	Website<br><br>**Excluded:** Wrong Population	Guideline (5)	https://designingfordementia.eu/resources/mind-guidelines

**19. Co-Design Toolkit**	Website<br><br>**Excluded:** Wrong Population	Toolkit (5)	https://aci.health.nsw.gov.au/projects/co-design/library-of-related-resources

**20. A Guide to Co-production for Researchers, Services and Commissioners**	PDF<br><br>**Excluded:** Wrong Population	Guide (5)	https://arc-kss.nihr.ac.uk/document-download/77-what-is-co-productionapril-2021/file
**21. The role of co-design in policy-making processes: lessons learnt from the CO3 Project**	PDF<br><br>**Excluded:** Wrong Population context & concept	Information (4)	https://www.projectco3.eu/wp-content/uploads/2020/07/policy_brief_02_2020_v2.pdf
**22. Family Engagement in Research: A conversation Guide**	PDF<br><br>**Excluded:** Context & concept	Engagement Guide (5)	https://canchild.ca/system/tenon/assets/attachments/000/003/637/original/Family_Engagement_in_Research__A_Conversation_Guide.pdf
**23. Come Together: Reasons to Include Participatory Design in Your**	Website<br><br>**Excluded:** Wrong Population context & concept	Information (4)	https://dscout.com/people-nerds/participatory-design

**Research Process**			
**24. Patient Engagement in Health Research: A How to Guide for Researchers**	PDF<br><br>**Excluded:** Wrong Population	Guide (5)	https://absporu.ca/wp-content/uploads/2020/05/How-To-Guide-ResearcherVersion-8.0-May-2018-1.pdf
**25. Guidelines**	Website<br><br>**Excluded:** Wrong Population	Guide (5)	https://www.disabilityinnovation.unsw.edu.au/inclusive-research/guidelines
**26. How we work together (Trainee Edition)**	PDF<br><br>**Excluded:** Context & concept	Casebook (4)	https://www.ktpathways.ca/system/files/resources/2020-12/IKTRNcasebook_vol-3.pdf
**27. Guides, toolkits and Reports**	Website<br><br>**Excluded:** Context & concept	Guide (5)	https://productivemargins.blogs.bristol.ac.uk/resources/guides/

**28. CoProduction: Research and Practice Review Full Report**	PDF<br><br>**Excluded:** Wrong Population context & concept	Report: Importance of (4)	https://www.sheffield.ac.uk/sites/default/files/2022-03/Coproduction%20report%20-%20Full%20Report.pdf

**29. CoProducing research**	Website<br><br>**Excluded:** Wrong Population	Information on IKT (3/4/5)	https://www.imperial.ac.uk/patient-experience-research-centre/ppi/coproducing-research/
**30. CoProduce: Valuing all experience**	Website (various linkages to guideline, education, and information on the website)<br><br>**Excluded:** Wrong Population	Information on IKT (3/4/5)	https://www.sheffield.ac.uk/co-produce/toolkits-and-techniques
**31. The Coproduction Kickstarter**	Website<br><br>**Excluded:** Wrong Population	Information (4)	https://www.uts.edu.au/about/faculty-health/news/co-productionkickstarter
**32. CoProduction Kickstarter**	PDF<br><br>**Excluded:** Wrong Population	Guide (5)	https://cmhdaresearchnetwork.com.au/wp-content/uploads/2022/03/CMHDARN_-CoProduction_Kickstarter_FINAL22.4.22.pdf

**33. Community Engaged Scholarship Toolkit**	Website<br><br>**Excluded:** Wrong Population	Guide (5)	https://www.usf.edu/engagement/faculty/community-engaged-scholarshiptoolkit.aspx
**34. Community Engaged Scholarship**	Website<br><br>**Excluded:** Wrong Population	Guide (5)	https://communityengagement4.sites.olt.ubc.ca/scholarlyresources/community-engaged-scholarship/
**35. Community-Engaged Scholarship**	PDF<br><br>**Excluded:** Wrong Population context & concept	Information (4)	https://louisville.edu/communityengagement/faculty/faculty_handbook92519
**36. Introductory guide to Engaged Research**	PDF<br><br>**Excluded:** Wrong Population context & concept	Information (4)	https://www.ru.ac.za/communityengagement/engagedscholarship/engagedresearch/

**37. Scholarship at the Heart of the City**	PDF<br><br>**Excluded:** Wrong Population context & concept	Strategic Plan for Research (1)	https://www.chairs-chaires.gc.ca/program-programme/srp-prs/macewaneng.pdf
**38. MRAP Working Paper: A Guide to Engaged Scholarship**	PDF<br><br>**Excluded:** Wrong context & concept	Resource (3)	https://static1.squarespace.com/static/595a99c378d171b197d28683/t/5c155b2221c67c11ef97487d/1544903459337/CBR+-+Guide.pdf

**39. Community-Based (Participatory) Research (CB(P)R)**	Website<br><br>**Excluded:** Wrong Population, Context & Concept	Information (4)	http://actioncatalogue.eu/method/7421
**40. What we should know about Community-Based Participatory Research**	Presentation<br><br>**Excluded:** Wrong Population context & concept	Education Session (3)	https://www.lacats.org/documents/About%20CBPR%2010-08-13.pdf

**41. The Essential Guide to Doing Your Research**	PDF<br><br>**Excluded:** Wrong Context & concept	Information (4)	https://www.ru.ac.bd/wp-content/uploads/sites/25/2019/03/402_06_00_O'Leary-The-Essential-Guideto-Doing-Your-Research-Project-2017.pdf
**42. Community-Engaged Research with Community-Based Clinicians**	PDF<br><br>**Excluded:** Wrong Population	Guide (5)	https://synergy.dartmouth.edu/sites/default/files/docs/CEnR_with_Community_Based_Clinicians_Resource_Manual_for_Researchers.pdf
**Targeted University Website**<br>*Specific documents available upon request, all documents have been accessed via listed university website<br>*** All documents were excluded for having the wrong population, context, and concept, unless otherwise indicated**			
**Title**	**Type**	**Topic (Theme)**	**Located at ____ university**

**43. Indigenous Engagement and Collaboration**	Course	Education (3)	University of Concordia Website
**44. Knowledge Mobilization (KMb) at CUE**	Event	Event (3)	University of Concordia Website

**45. Seed Grant**	Grant	Grant (2)	University of Concordia Website
**46. Funding Opportunity – SSHRC Knowledge Synthesis Grants**	Grant Application	Grant (2)	University of Concordia Website
**47. Internal Research Grants – Impact Stream**	Grant	Grant (2)	University of Concordia Website
**48. Internal Research Grants- Seed Stream**	Grant	Grant (2)	University of Concordia Website
**49. Insight Development Grants**	Grant / Presentation	Grant (2/3)	University of Concordia Website

**Funding Opportunity**			

**50. Creating Pathways into the Economy Tomorrow**	Website	Information (4)	University of Concordia Websites
**51. BMO Centre for Innovation and Applied Research**	Program	Education (3)	University of Concordia Website
**52. Annual Report 2019/2020**	Report PDF	Strategic Reports and Plans (1)	University of Concordia Website
**53. Strategic Research Plan 2019-2024**	Report PDF	Strategic Report (1)	University of Concordia Website
**54. Comprehensive Institutional Plan 20152018**	Report PDF	Strategic Report (1)	University of Concordia Website
**55. Comprehensive Institutional Plan 20192022**	Report PDF	Strategic Report (1)	University of Concordia Website

**56. Research at Concordia**	Website	Information (4)	University of Concordia Website
**57.Annual Report 2018/2019**	PDF	Strategic Plan (1)	University of Concordia Website
**58. Knowledge Utilization Colloquium 2011**	PDF	Event (3)	University of Alberta
**59. Community Based Participatory Action Research**	Presentation	Event (3)	University of Alberta
**60. Knowledge Utilization Colloquium '01**	Event Debrief	Event (3)	University of Alberta

**61. Creating Collaborative, Shared Research Initiatives: Strategies and Approaches**	Education Presentation	Education (3)	University of Alberta

**62. Casting fresh light on the 'Shining Mountains'**	Report	Information (4)	University of Alberta
**63. Mentorship and Training to strengthen Indigenous research capacity**	Website	Education Resource (3)	University of Alberta
**64. Speeding up the Spread**	PDF	Information (4)	University of Alberta
**65. The Road Ahead: Knowledge Bank Progress Report**	PDF	Information (4)	University of Alberta

**66. AbSPORU Faculty of Medicine and Dentistry Webinar**	Presentation	Education/ Grant (2/3)	University of Alberta
**67. Alberta SPOR SUPPORT Unit (AbSPORU) Graduate Studentship in Patient Oriented Research**	Website	Grant (2)	University of Alberta
**68. AHS Strategy for Clinical Health Research Innovation and Analytics 2015-2020**	PDF	Strategic Plan (1)	University of Alberta
**69. Community Engagement**	Website<br><br>**Excluded:** Wrong Population	Information/ Guide (4)	University of Alberta

**70. Community Engagement Plan**	PDF & Plan<br><br>**Excluded:** Wrong Population	Guide (5)	University of Alberta
**71. Resiliency Resolve Results**	Handbook	Information (4)	University of Alberta
**72. Families First Edmonton: Putting the Research to Work in 2010-2012**	Project Charter/Plan	Information (4)	University of Alberta

**73. Kias Annual Report**	Report	Strategic Plan (1)	University of Alberta
**74. Comprehensive Institutional Plan**	Plan	Strategic Plan (1)	University of Alberta
**75. Master of Nursing Curriculum Guide**	Plan	Strategic Plan (1)	University of Alberta

**76. Grad Student Collaborating with First Nation on Governance Project**	Website	Information (4)	University of Calgary
**77. Mobilizing Knowledge On Newcomers Symposium**	Event	Event (3)	University of Calgary
**78. Cultural Protocol at UCalgary**	Website	Information (4)	University of Calgary
**79. Program Guide: Clinician Researcher**	Program	Education (3)	University of Calgary

**Training Stream**			
**80. Academic Plan 2012**	Strategic plan	Strategic Plan (1)	University of Calgary

**81. Energizing Eyes High Strategy 20172022**	Environmental Scan of plans	Strategic Plan (1)	University of Calgary
**82. Institutional Sustainability Strategy**	Presentation	Strategic Plan (1)	University of Calgary
**83. 2012 Strategic Research Plan**	Presentation	Strategic Plan (1)	University of Calgary
**84. CANet Strategic Research Grant**	Grant Application	Grant (2)	University of Calgary
**85. Natural Sciences and Engineering Research Council Collaborative Research and Development Grants | 2015**	Grant Application	Grant (2)	University of Calgary

**86. SPOR Networks – Knowledge Mobilization and Implementation Science**	Grant Announcement	Grant (2)	University of Calgary
**87. Team Grant: CRISM Phase 2: Regional Nodes**	Grant Announcement	Grant (2)	University of Calgary
**88. Finding a Place within CIHR: A guide for social sciences and humanities health researchers**	Presentation	Grant (2)	University of Calgary
**89. Strategic Research Plan**	Strategic Research Plan	Strategic Plan (1)	University of Lethbridge
Documents 90-105 were unable to be retrieved.			
**Location of fieldnotes**	**Theme based on**	**Additional Comments Document**	

Simon Fraser	Guide for Researchers (5)	
University of Manitoba	Information (4)	Knowledge Translation Template
University of Manitoba	Guide for Researchers (5)	
University of Guelph	Guide to IKT (5)	
University of Guelph	Guide to IKT (5)	
University of Waterloo	Information (4)	
University of Waterloo	Information (4)	
University of Waterloo	Information (4)	
University of Windsor	Information/Strategic Plan (1)	PhD Handbook

York University	Information (4)	
York University	Information (4)	
York University	Information (4)	
York University	Information (4)	
York University	Information (4)	
York University	Information (4)	
University of Regina	Education (3)	Education toolkit
